# Association of Prx4, Total Oxidant Status, and Inflammatory Factors with Insulin Resistance in Polycystic Ovary Syndrome

**DOI:** 10.1155/2021/9949753

**Published:** 2021-06-22

**Authors:** Sahar Mazloomi, Nasrin Sheikh, Marzieh Sanoee Farimani, Shamim Pilehvari

**Affiliations:** ^1^Department of Clinical Biochemistry, Faculty of Medicine, Hamadan University of Medical Sciences, Hamadan, Iran; ^2^Students Research Committee, Hamadan University of Medical Sciences, Hamadan, Iran; ^3^Department of Obstetrics and Gynaecology, Medicine School, Hamadan University of Medical Sciences, Hamadan, Iran; ^4^Endometrium and Endometriosis Research Center, Hamadan University of Medical Sciences, Hamadan, Iran

## Abstract

**Introduction:**

Chronic inflammation and oxidative stress conditions have been reported in women with polycystic ovary syndrome (PCOS). Peroxiredoxin 4 (Prx4) is a related antioxidant in insulin synthesis. We hypothesized that insulin resistance in these women is associated with total oxidant status (TOS) and inflammatory factors.

**Materials and Methods:**

Two hundred three people including 104 PCOS patients and 99 healthy women, who were matched for age and body mass index (BMI), entered the study. Waist circumference of the participants was measured; serum glucose, lipid profile, insulin, Prx4, TOS, hs-CRP, and TNF-*α* were also evaluated.

**Results:**

The Prx4 level was significantly lower in PCOS than in the control group. In addition, marked increase was observed in the concentration of TOS, hs-CRP, and TNF-*α* in PCOS, compared to the healthy women. There was a positive correlation of TOS with hs-CRP, TNF-*α*, and HOMA-IR. The risk of PCOS for subjects with high hs-CRP was 60 times greater than those who had low serum hs-CRP concentration; after performing multiple logistic regression analyses with the backward method, TNF-*α* was considered as an effective biomarker to predict PCOS *β* = 49.087 (all *p* < 0.05).

**Conclusion:**

This study identified increased oxidative stress and inflammation in PCOS; this may be due to decrease in the antioxidants, such as Prx4.

## 1. Introduction

Polycystic ovary syndrome (PCOS) is the most common cause of infertility, with a prevalence of approximately 9–18% in women in the childbearing age [1]. PCOS is characterized by symptoms such as clinical or biochemical hyperandrogenism, chronic anovulation, and polycystic ovaries; it is widely associated with insulin resistance. This disease is a metabolic disorder, in which insulin resistance and hyperinsulinemia are seen [2]. Insulin resistance has been reported in 50–70% of women with PCOS; it is now identified as an important risk factor to develop metabolic syndrome in the cited women [3, 4].

Recently, studies have shown that inflammation is involved in ovulation. It can be caused by visceral adipose tissue; fat cells can maintain the inflammation by elevating the production of inflammatory cytokines. Due to the prevalence of obesity in PCOS, low-grade inflammatory conditions and inflammatory markers are detectable [5–8]. In PCOS patients, compared to healthy women, the level of inflammatory factors such as C reactive protein (CRP), tumor necrosis factor-*α* (TNF-*α*), and interleukins (IL-1, IL-6, and IL-18) increases [9, 10]. TNF-*α* is a proinflammatory cytokine, which is secreted by granulose-luteal cells, ovarian macrophages, and immune cells [11]; it interferes in inflammation and immune responses [12]. TNF-*α* has a role in PCOS patients with obesity and hyperandrogenism [13].

In addition to mild chronic inflammation, oxidative stress can also play a role in PCOS and its metabolic processes. Oxidative stress is caused by antioxidants imbalance due to the uncontrolled formation of oxidants in the limited state of antioxidant defense system [10, 14, 15]. Preventive and restorative mechanisms are physiological protective measures against a wide range of oxidants [16]. Antioxidants are able to sweep, excrete, or inhibit the formation of oxidants. They include enzymes such as catalase, superoxide dismutase (SOD), peroxiredoxin (Prx), glutathione peroxidase (GPX), and paraoxonase and small molecules such as glutathione, vitamin *E*, and ascorbic acid [17].

Peroxiredoxins (Prxs) were first identified in 1994, as a new class of peroxidase; they belong to the family of cysteine-dependent peroxidase enzymes. They scavenge peroxynitrite and peroxide in the mammalian cells [18, 19]. Prxs have six members; they can remove more than 90% of cellular peroxides [20]. These isozymes are distributed among different organelles, such as nucleus, cytosol, mitochondria, peroxisomes, and endoplasmic reticulum (ER).

Prxs control cytokine-induced peroxide amount; thus, they regulate signal transduction of mammalian cells. Prx4 is located in the ER [21].

The role of isoforms of Prxs as a marker of oxidative stress has been seen in some diseases; isoform 4 (Prx4) has been introduced as a diagnostic marker of oxidative stress in diabetes [22–24]. Studies have shown that Prxs and Prx4 protect cells from oxidative stress, which is directly related to persistent inflammatory markers, including procalcitonin, CRP, and IL-6 [25, 26]. An association has been observed between Prx4 and insulin secretion [27, 28]; also, Prx4 has been introduced as a new biomarker of oxidative stress in cardiovascular diseases [25]. Decreased serum level of Prx4 has been observed in PCOS, as well as granulosa cells in women with PCOS [29, 30].

Recently, many studies have been conducted on the role of oxidative stress in the development of insulin resistance, diabetes, and cardiovascular disease [31–33]. Since the prevalence of these cases is high in women with PCOS [34], therefore, the review of possible shortage in various antioxidants in creation and progression of PCOS is important. To the best of our knowledge, limited studies have investigated the association of Prx4 and inflammatory factors in PCOS. Therefore, the aim of this study was to investigate plasma levels of Prx4, inflammatory factors, and total oxidant status to evaluate the relationship between these factors and insulin resistance in PCOS subjects.

## 2. Materials and Methods

### 2.1. Subjects

A total of 203 women, including 104 newly diagnosed PCOS cases and 99 healthy women as control, were serially enrolled in this study. The age range of whole studied individuals was 18–38 years. All of the subjects were outpatients at the Gynecology and Obstetrics Clinic of Fatemieh Hospital of Hamadan University of Medical Sciences; they were diagnosed with PCOS, based on the Rotterdam criteria, by two of the following three features: oligo- or anovulation, clinical and/or biochemical signs of hyperandrogenism, or polycystic ovaries [35]. All the subjects with secondary PCOS, resulting from conditions such as prolactinoma, congenital adrenal hyperplasia, Cushing syndrome, and virilizing ovarian or adrenal tumors, were excluded from the study. Control subjects were selected from the same socioeconomic population; they were matched for age and body mass index (BMI) to the affected cases. All women in the control group had normal ovulation cycles and no signs of hyperandrogenism. None of the women had any systemic disease, nor did they use any medication that might affect their reproductive physiology. Approval was obtained from the ethics committee of Hamadan University of Medical Sciences (ethics committee code: IR.UMSHA.REC. 1399.328).

### 2.2. Measurement

The weight of studied individuals was measured with a balanced-beam scale to the nearest 0.1 kg, while wearing light clothing. Height was also measured with a stadiometer to the nearest 0.5 cm. BMI was calculated based on the weight/(height)2 formula. Waist circumference between the lowest rib and the iliac crest was measured at the level of umbilicus, two times using flexible tape.

Blood samples were collected between 8:00 and 9:00 a.m. on days 3–6 of the spontaneous menstrual cycle, after at least 12 hours of fasting. The basal levels of Prx4, hs-CRP, TNF-*α*, total oxidant status (TOS), insulin, serum glucose (Pars Azmoon, Iran), lipid profile (Pars Azmoon, Iran), luteinizing hormone (LH), and follicle-stimulating hormone (FSH) were measured. The homeostasis model assessment index (HOMA index) was used to determine the level of insulin resistance; it was calculated according to the following equation: (insulin (*μ*U/mL)) × (fasting blood sugar (FBS) (mmol/L))/22.5. In the present study, insulin resistance was defined by the HOMA index of more than 2.1 [36].

Insulin level, LH, and FSH concentration were measured via an electrochemiluminescence immunoassay (ECLIA), using commercially available kits (Roche, Germany).

### 2.3. Peroxiredoxin 4

It was assessed by a ready-to-use ZellBio-GmbH ELISA Kit (Cat. ZB-12927C–H9648, Ulm, Germany); the kit was used according to the manufacturer's instructions. Cited measurement is based on the noncompetitive Sandwich method. The standard range for Prx4 concentration was 1.25–20 ng/ml.

### 2.4. Total Oxidant Status (TOS)

TOS concentration was assessed using a ready-to-use Natos Kit (Navand Salamat Co., Iran). The assay is based on the oxidation reaction, taking place by amplifying molecules, which are present in large quantities in the reaction medium. The color intensity, which can be measured by spectrophotometry, is related to the total amount of oxidant molecules in the sample. This test is calibrated with hydrogen peroxide, and results are expressed in terms of equivalent liquid peroxide per liter (*μ*mol H_2_O_2_ Equiv/L). The standard range for TOS concentration was 0.156–10 *μ*mol Equiv/L, and the sensitivity range was also 0.023 *μ*mol Equiv/L.

### 2.5. hs-CRP

High-sensitivity C-reactive protein (hs-CRP) concentration was assessed using a ready-to-use Monobind ELISA Kit (Cat. 3125-300A, USA), according to the manufacturer's instructions. The basis of the measurement is enzyme immunoassay and colorimetric streptavidin biotin based Sandwich assay method; the sensitivity was also 0.014 µg/ml.

### 2.6. TNF-*α*

TNF-*α* level was measured using a ready-to-use Demeditec ELISA Kit (Germany), according to the manufacturer's instructions. The basis of the measurement is TMB colorimetric Sandwich ELISA kit, with short assay time detecting TNF-*α* with 0.7 pg/ml sensitivity; the standard range for TNF-*α* concentration was 5.35–530 pg/ml.

### 2.7. Statistical Analysis

Data analysis was performed using SPSS software version 20.0 (SPSS Inc., Chicago, IL, USA) and Graph Pad Prism software version 8.0 (Graph Pad Software, CA, USA). Kolmogorov-Smirnov test, Mann–Whitney U test, and Student's *t*-test were used as appropriate. To assess the relationship between the variables, Spearman's correlation coefficient (*r*) was used. Multiple logistic regression analyses (univariate and with backward method) were used to assess the independent effect of variables on the odds for PCOS. Data were presented as means ± SE. The statistical significance was set at *P* ≤ 0.05.

## 3. Results

### 3.1. Demographic Data and Biochemical Parameters

The demographic results showed that the minimum and maximum age of participants were 18 and 38 years, respectively; the mean age was 26 years with no statistical difference between the two groups. The demographic results are presented in Table 1. The groups were also matched for age (*p*=0.775) and BMI (*p*=0.420). Although the two groups were similar in terms of BMI, waist circumference was higher in PCOS group (*p*=0.003). Although lipid parameters such as cholesterol, TG, and low-density lipoprotein (LDL) was higher in PCOS groups (*p* < 0.05), the level of high-density lipoprotein (HDL) in the two groups was similar (*p*=0.261). There was also no significant difference in FBS concentration (*p*=0.163). LH/FSH ratio, insulin concentration, and HOMA-IR were significantly higher in women with PCOS (*p* < 0.001).

### 3.2. Prx4, TOS, hs-CRP, and TNF-*α* Analysis

**Figure 1:**
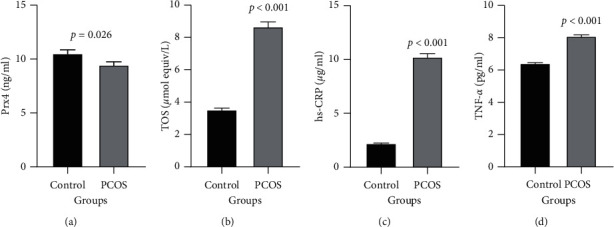
Comparison of Prx4 (a), TOS (b), hs-CRP (c), and TNF-*α* (d) concentrations in two groups of control and PCOS. Two group variables show a normal distribution and are given as mean ± SE.

The level of Prx4 was significantly lower in PCOS 9.45 ± 0.302 ng/ml compared to the control group 10.52 ± 0.337 ng/ml (*p*=0.026). The mean TOS level was different in the patient group compared to the controls (8.66 ± 0.299 versus 3.52 ± 0.116 *μ*mol Equiv/L, respectively, *p* < 0.001). A significantly higher level of serum hs-CRP was found in PCOS 10.23 ± 0.332 *μ*g/ml, compared to the control group 2.22 ± 0.066 *μ*g/ml (*p* < 0.001), and the level of TNF-*α* was different in PCOS 8.09 ± 0.092 *μ*g/ml, compared to the control group 6.41 ± 0.063 *μ*g/ml (*p* < 0.001), as shown in Figure 1.

### 3.3. Correlation Analysis of TOS with Prx4, hs-CRP, TNF-*α*, and Insulin Serum Levels

**Figure 2:**
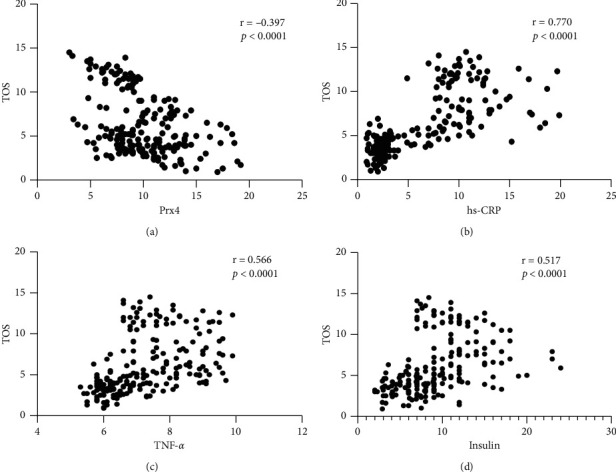
Correlation analysis of TOS with Prx4, hs-CRP, TNF-*α* serum levels, and HOMA-IR. According to correlation analysis, the TOS variable had a negative association with Prx4 (a), but there was a positive correlation between TOS level and hs-CRP (b), TNF-*α* (c), and insulin (d). Spearman's correlation coefficient (*r*) was used to examine the relationship between the variables.

According to correlation analysis, the TOS variable had a significant negative association with only Prx4 (*p* < 0.0001, *r* = ─0.397); TOS had a significant positive association with hs-CRP (*p* < 0.0001, *r* = 0.770), TNF-*α* (*p* < 0.0001, *r* = 0.566), and insulin (*p* < 0.0001, *r* = 0.517) (Figure 2).

**Figure 3:**
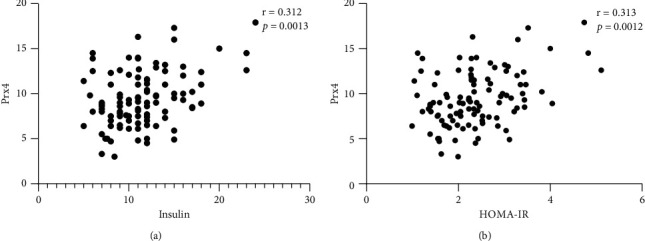
Correlation analysis of Prx4 with insulin serum level and HOMA-IR in the PCOS group. According to correlation analysis, the Prx4 variable had a positive association with insulin (a) and HOMA-IR (b). Spearman's correlation coefficient (*r*) was used to examine the relationship between the variables.

Although no significant correlation was seen between Prx4 and insulin and HOMA-IR, interestingly there was a significant positive relationship between Prx4 and insulin and HOMA-IR in PCOS group (*p* > 0.05) (Figure 3). Moreover, there was an inverse correlation between Prx4 and BMI in PCOS group (*p*=0.013, *r* = -0.243).

### 3.4. Increased Chance of PCOS by Elevating Inflammatory Factors

The results of logistic regression analysis of each variable showed that increasing inflammatory factors is more effective in causing disease than increasing TOS levels and decreasing Prx4 antioxidant (Table 2). In the next step, logistic regression of backward method determined that only TNF-*α* (OR = 49.087, *p* < 0.001) and TOS (OR = 9.386, *p* < 0.001) affect PCOS (Table 3).

### 3.5. hs-CRP as a Biomarker in PCOS

The results of the ROC curve show that the increase in hs-CRP (AUC = 0.999, *p* < 0.001) compared to TNF-*α* (AUC = 0.772, *p* < 0.001) as an excellent biomarker has detection power.

## 4. Discussion

PCOS is a multifactorial disorder diagnosed by hyperandrogenism, polycystic ovary, and chronic anovulation in premenopausal women [2]. Despite extensive studies about the pathogenesis of PCOS, the cause is not determined yet. In recent years, several causative hypotheses have been proposed for PCOS, such as insulin resistance, chronic inflammation, oxidative stress, family history, and genetics [15, 37, 38]. In addition to antioxidant property of PRX4, it is also involved in the process of insulin synthetization and secretion [27, 28]. The findings of the present study showed that the serum levels of Prx4 were lower in women with PCOS compared to the healthy control group. In this regard, the amount of inflammatory factors (TNF-*α* and hs-CRP) increased significantly (*P* < 0.05).

In line with our study, the results of a study by Gateva et al. showed a decrease in Prx4 levels in women with PCOS. The amount of Prx4 in nonobese patients with PCOS was significantly lower than obese patients with and without PCOS [29]. The effect of excess androgens on the production of oxidative stress factors has been reported [39]. Since one of the main causes of PCOS is the increase of androgen levels, so PCOS itself can cause oxidative stress and reduce antioxidants such as Prx4. However, further studies are needed to examine the mechanism of Prx4 as the antioxidant defense in PCOS. The results of the study by Kordestani et al. showed that the level of TNF-*α* in PCOS was higher than healthy women. Furthermore, a positive correlation was reported between the level of TNF-*α* and insulin resistance. Our results showed that serum TNF-*α* levels were significantly higher in PCOS women, as well as hs-CRP levels. There was also an association between the level of inflammatory factors and insulin resistance. Our findings showed that TNF-*α* level was significantly associated with Prx4. The presence of chronic inflammation may activate this defense process by affecting the antioxidant system [13]. Chronic inflammatory markers enhance insulin resistance and hyperandrogenism; therefore, those are involved in the pathogenesis of PCOS [40]. In a review study by Yamada et al. in Japan, Prx4 was reported to exert its protective function against oxidative damage by inhibiting reactive oxygen species (ROS) in the extracellular space. Prx4 was also associated with inflammation and insulin resistance related diseases, such as diabetes and atherosclerosis. In this regard, our study showed a significant correlation between Prx4 levels and TNF-*α*; in line with the antioxidant role of Prx4, an inverse correlation was observed between the Prx4 level and TOS amount [41].

Gateva et al. found that prediabetes patients had higher concentration of Prx4; this difference was not observed in people with or without insulin resistance. In contrast, our results showed an association between Prx4 and insulin resistance in PCOS women although we found no association with glucose levels. Differences in the disease type and the number of samples may be the cause of difference in results [42].

The results of the present study showed that the level of hs-CRP in women with PCOS increased significantly; it was in line with the results of Mažibrada and colleagues. They found that hs-CRP levels were significantly higher in young girls with PCOS in comparison to the healthy girls. These findings suggest that chronic inflammation is involved in the pathophysiology of PCOS [43]. Similar to our findings, Sadeghi et al. also showed higher level of hs-CRP and insulin resistance in PCOS, compared to the control group [44]. The majority of studies addressing the status of chronic low grade inflammation in PCOS has focused on the measurement of circulating C-reactive protein (CRP), TNF-*α*, IL-18, and IL-6 [45]. In PCOS, increases in NF*κ*B activation and circulating CRP and decreases in I*κ*B*α* protein following saturated fat ingestion are independent of obesity [46]. On the other hand, increased CRP levels are positively correlated with insulin resistance and the incidence of T2DM. Therefore, high CRP concentrations are considered a potential cause of long-term consequences of PCOS [47].

In the study by Bañuls et al., ROS level of polymorphonuclear leukocytes (PMNs) was examined. The results of their study showed higher level of ROS in PCOS individuals, and association between altered metabolic status, increased ROS production, ER stress, and leukocyte–endothelium interactions in PCOS was observed, all of which are related to vascular complications [48]. In the present study, we measured the TOS level; the results showed that the level of TOS in women with PCOS was significantly higher than the control group. Studies on the level of oxidative stress in the follicular fluid of women with PCOS have shown that the levels of TOS and TNF-*α* in the follicular fluid of these women are significantly increased compared to the control group [49, 50]. Furthermore, the ROS level in granulosa cells derived from PCOS women was significantly higher than non-PCOS granulosa cells [51]. There are several sources that generate the ROS; one source of reactive oxygen under normal conditions in humans is the leakage of activated oxygen from mitochondria during oxidative phosphorylation. Another endogenous source of ROS is the leakage of activated oxygen from the detoxification reactions involving the liver cytochrome P-450 enzyme system [45]. Aromatase is a cytochrome P-450 hemoprotein-containing enzyme complex that catalyzes the conversion of androstenedione and testosterone into oestrogens. On the other hand, aromatase function is defective in PCOS [52]. Mitochondrial dysfunction has also been observed in PCOS following increased androgens and insulin resistance [53–55].

Jatzko and her colleagues found that CRP was significantly higher in PCOS patients. They also believed that elevated CRP in PCOS is independent from obesity [56]. However, Sharifi et al. did not observe any significant differences in CRP level between women with PCOS and the control group. Probably the inflammatory protein concentration measurement range made this difference; in the present study, hs-CRP was measured with high sensitivity. Insulin levels were significantly higher in women with PCOS, as well as insulin resistance in both studies [57]. Citing the previous researches, our results also indicated the presence of oxidative stress, as well as inflammation in PCOS. In a review study by Cozzolino et al. in the USA and Spain, it was reported that an increase in ROS in PCOS patients is associated with insulin resistance. In this regard, our results also showed a direct relationship between the amount of TOS and insulin resistance [58].

Meng et al. demonstrated that Prx4 as an antioxidant was expressed at lower levels in the polycystic ovaries compared to the normal ovaries; they also reported higher expression of Prx4 in the granulosa cells of mature follicles compared to the GCs of immature follicles. Our results showed a significant decrease in serum Prx4 levels. In general, these findings indicated a decrease in the Prx4, as an important antioxidant at the cellular and serum levels, in this disease [30].

The present study showed the inverse correlation of TOS and Prx4 in women with PCOS, for the first time. Some effective limitations can be mentioned in our study. This study was performed with a relatively small sample size of patients, and therefore confirmation of our findings requires studies with a larger sample size. Another limitation was the lack of measurement of Prx4, TNF-*α*, and hs-CRP gene expression. We believe that separate determination of oxidative status and other antioxidant forms and measurement of inflammatory cytokines may strengthen the results.

In conclusion, the results of this study together with the findings of previous researches confirmed the role of oxidative stress and inflammation in PCOS pathogenesis and showed its effect on insulin resistance. In addition, changes in the level of antioxidants, such as Prx4, indicated that special attention should be paid for controlling the antioxidants, in addition to the main therapeutic strategy in PCOS women.

## Figures and Tables

**Table 1:** Biochemical indexes and clinical characteristics of the women with PCOS and the control group (mean ± SE).

Variable	Control group (*n* = 99)	PCOS group (*n* = 104)	*p* value
Age	26.05 ± 0.487	25.82 ± 0.408	0.775
BMI^b^	26.43 ± 0.384	26.84 ± 0.341	0.420
Waist	86.44 ± 1.100	91.57 ± 1.090	0.003
TC	162.99 ± 3.444	172.63 ± 3.319	0.044
TG	124.18 ± 2.308	151.21 ± 5.721	0.002
LDL	92.76 ± 2.264	101.34 ± 2.161	0.007
HDL	48.77 ± .0941	47.33 ± 0.916	0.261
FBS	82.75 ± 0.722	84.03 ± 0.571	0.163
LH/FSH	1.37 ± 0.091	3.5 ± 0.355	<0.001
Insulin	7.02 ± 0.331	11.41 ± 0.377	<0.001
HOMA-IR	1.45 ± 0.074	2.37 ± 0.081	<0.001

BMI: body mass index; TC: total cholesterol; TG: triglyceride; LDL: low-density lipoprotein; HDL: high-density lipoprotein; FBS: fasting blood sugar; FSH: follicle-stimulating hormone; LH: luteinizing hormone.

**Table 2:** Univariate logistic regression model of hs-CRP and other confounding variables to predict PCOS.

Dependent variables	Independent variables	Odds ratio (Exp (*β*))	CI for Exp (*β*)	*p* value
PCOS	hs-CRP	60.988	3.212–1157.952	<0.006
	TNF-*α*	13.455	6.637–27.280	<0.001
	TOS	5.234	3.705–8.910	<0.001
	Prx4	-0.902	0.826–0.985	0.022

hs-CRP: high-sensitivity c-reactive protein; TNF-*α*: tumor necrosis factor; TOS: total oxidant status; Prx4: peroxiredoxin 4.

**Table 3:** Multiple logistic regression model of TNF-*α*, TOS, and other confounding variables to predict PCOS.

Dependent variables	Independent variables	Odds ratio (Exp (*β*))	CI for Exp (*β*)	*p* value
PCOS	TNF-*α*	49.087	6.752–356.887	<0.001
	TOS	9.386	3.102–28.402	<0.001

## Data Availability

The demographic data and biochemical parameters used to support the findings of this study are included within the article.

## Additional Points

*Highlights.* (1) Inflammatory elements such as hs-CRP and TNF-*α* are markedly higher in PCOS. (2) hs-CRP is probably an important biomarker in PCOS diagnosing. (3) Increased total oxidant status is seen in PCOS. (4) Prx4 is significantly lower in women with PCOS.
